# Nature-inspired architected materials using unsupervised deep learning

**DOI:** 10.1038/s44172-022-00037-0

**Published:** 2022-11-25

**Authors:** Sabrina Chin-yun Shen, Markus J. Buehler

**Affiliations:** 1 Laboratory for Atomistic and Molecular Mechanics (LAMM), Massachusetts Institute of Technology, 77 Massachusetts Ave., Cambridge, MA 02139 USA; 2 Department of Materials Science and Engineering, Massachusetts Institute of Technology, 77 Massachusetts Ave., Cambridge, MA 02139 USA; 3 Center for Computational Science and Engineering, Schwarzman College of Computing, Massachusetts Institute of Technology, 77 Massachusetts Ave., Cambridge, MA 02139 USA

**Keywords:** Mechanical engineering, Materials science, Engineering

## Abstract

Nature-inspired material design is driven by superior properties found in natural architected materials and enabled by recent developments in additive manufacturing and machine learning. Existing approaches to push design beyond biomimicry typically use supervised deep learning algorithms to predict and optimize properties based on experimental or simulation data. However, these methods constrain generated material designs to abstracted labels and to “black box” outputs that are only indirectly manipulable. Here we report an alternative approach using an unsupervised generative adversarial network (GAN) model. Training the model on unlabeled data constructs a latent space free of human intervention, which can then be explored through seeding, image encoding, and vector arithmetic to control specific parameters of de novo generated material designs and to push them beyond training data distributions for broad applicability. We illustrate this end-to-end with new materials inspired by leaf microstructures, showing how biological 2D structures can be used to develop novel architected materials in 2 and 3 dimensions. We further utilize a genetic algorithm to optimize generated microstructures for mechanical properties, operating directly on the latent space. This approach allows for transfer of information across manifestations using the latent space as mediator, opening new avenues for exploration of nature-inspired materials.

## Abstract (web summary)

Shen and colleagues reported an unsupervised generative adversarial network (GAN) to identify patterns in leaves associated with superior mechanical properties and use 3D printing to build architected materials inspired by the patterns. In the future, this approach may be applied more broadly to natural materials to enable efficient algorithmic construction of structures with customized properties and form factors.

## Introduction

Natural materials have been optimized over millions of years of evolution, creating a rich source of design inspiration for modern engineering. Biological materials are largely composed of organic and ceramic materials, which are typically considered to be weaker than metals. Even so, evolution has succeeded in creating materials that are both strong and tough out of these basic building blocks by architecting at multiple scales^1–4^. Resultant architected and hierarchical materials, such as lamellar structures in conch shells^5,6^ or beta-sheet crystals in spider silk^7^, can have mechanical properties rivaling those of steel and certainly surpassing their synthetic counterparts^8,9^. This motivates the nature-inspired design paradigm: innovation can be accelerated by harnessing nature’s design principles through biomimicry and beyond and by exploiting these principles in novel designs further optimized for societal needs.

Manufacturing of complex architected materials has been enabled by recent developments in additive manufacturing technology, and hierarchical nature-mimicking materials have subsequently been created for diverse applications^10–15^. In the next generation of nature-inspired materials, machine learning is often used to push bioinspiration beyond mimicry without brute force computation, harnessing underlying design principles in nature toward specific applications. Algorithms such as multilayer perceptrons (MLPs), convolutional neural networks (CNNs), recurrent neural networks (RNNs), gaussian processes (GP), and genetic algorithms have been used to predict and optimize properties including strength^16,17^, toughness^17,18^, failure mode^19^, yield stress, and yield strain^20^. While these methods are powerful for optimizing within a set of constraints, they are limited by human intervention in data labeling, which defines predetermined design spaces. Further, they yield “black box” outputs that can only be indirectly manipulated through changes in machine learning architecture, training parameters, or cost functions. Thus even while requiring immense amounts of experimental or simulation data to train, each model is severely restricted in its application space. Alternatively, unsupervised generative methods like generative adversarial networks (GANs) make use of unlabeled data and, once trained, can produce infinite novel outputs^21^. The development of a latent space that can be explored, enables the generation of new designs without user input constraints, pushing the envelope of human understanding in design inspiration. These designs can also be tested, scored, and manipulated for functionality in various applications.

GANs train via pseudo-supervised learning tasks: two distinct internal neural networks, a generator and a discriminator, are trained against each other in a zero-sum game such that the generator becomes skilled at generating convincing samples as the discriminator becomes skilled at distinguishing between real input data and artificially generated data. This architecture allows the training of generative models for various fields, including domain-specific data augmentation, generation of art or life-like images, and image-to-image translation^21,22^.

The generator model in a GAN operates by seeding the generative algorithm with a fixed-length vector drawn randomly from a Gaussian distribution. The multidimensional vector space (say *N*-dimensional) from which it is drawn functions as a latent space onto which, after training, each piece of data from the original distribution can be mapped as a single point defined by an *N* × 1 vector. In this way, the GAN learns features of the original dataset and the latent space serves as a compression of relevant data distributions. Then, in generative steps, new points in latent space can be constructed into higher dimensional outputs^23^ in the form of the training data (novel images, for example), and the latent space becomes an infinite design space for novel samples. This makes GANs a powerful method for drawing desirable features and distributions from existing data and recomposing them into new data, such as generating deceptively realistic portraits of human faces^24^.

**Fig. 1:**
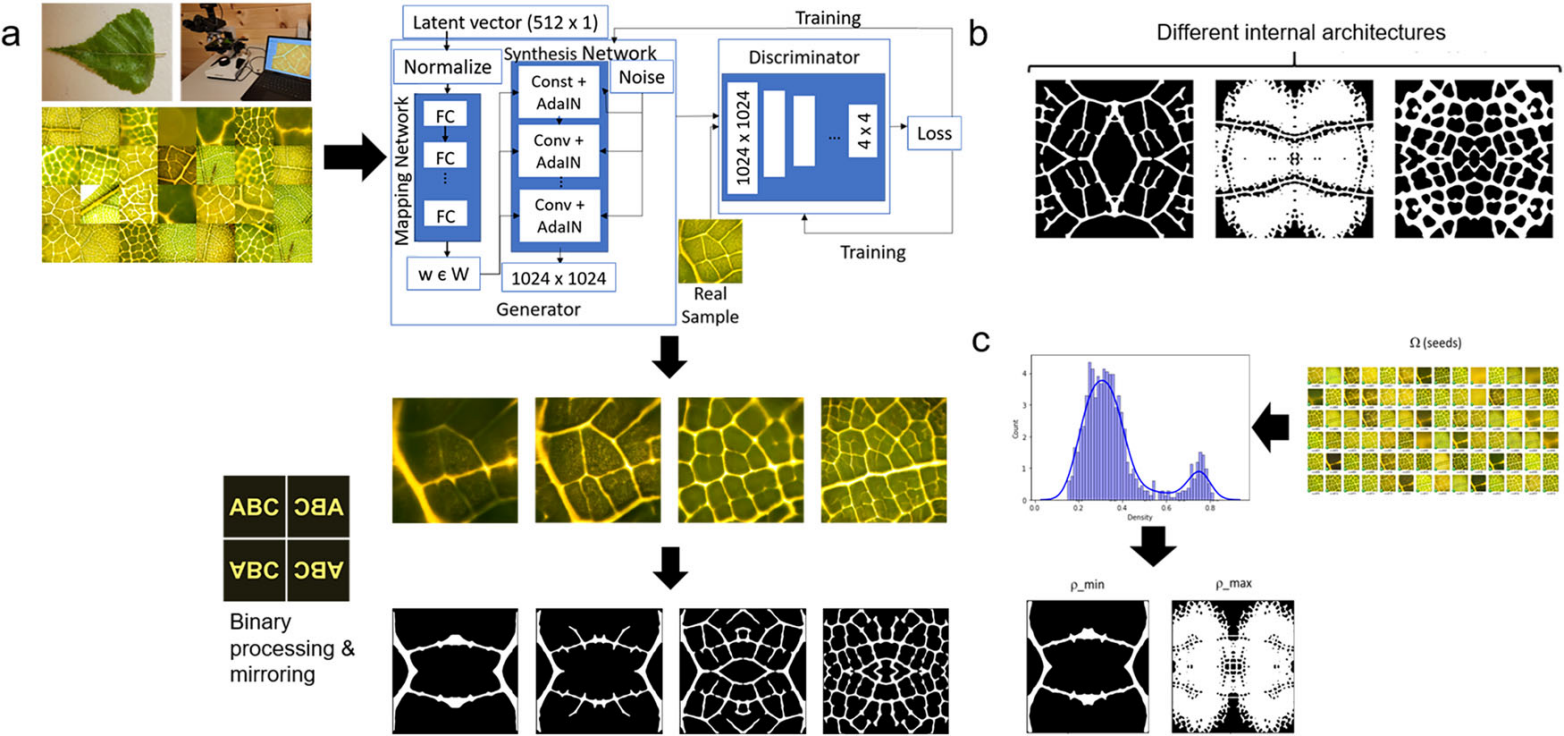
Generative adversarial network (GAN) training and processing of outputs. **a** Overview of the leaf image generation and dataset, including GAN implementation and image generation process. De novo leaf-based “unit cells” are produced via binary processing, island removal, and XY-mirroring of individual generated images. Examples shown represent continuously varying microstructures that can be derived from the latent space. Supplementary Movie 1 provides further insight into such variations. **b** Representative images for the three major structural families found in the generated leaf-inspired architectures. **c** Randomly seeded leaf images can be mined for specific properties, such as low or high density, which corresponds to mechanical properties.

GANs can be successfully applied to materials engineering. For example, a GAN was trained to generate over 400 2D material architectures that approach theoretical upper bounds on composite moduli^25^. Here we apply GANs to bio-inspired design for the first time using StyleGAN, a generative image modeling network constructed to enable scale-specific feature control, and therefore especially equipped for the design of hierarchical structures such as those found in nature ^26^. StyleGAN uniquely operates with both the *z*-latent space typical of a GAN and an intermediate *w*-latent space, which is calculated from *z* with a learned mapping network (structure shown in Fig. 1a). The *w*-vector is input into the generator at multiple different layers, enabling disentanglement of features. As such, different w-vectors can also be input into different layers of the generator to generate “mixed” outputs^24,27^. The expanded latent space created represents an expansive design space that encompasses all structural cues from the training data and potentially beyond. In addition to unique designs, continuously evolving architectures can be generated and stacked through small steps in the continuous latent space to build varying or three-dimensional materials, which can be further modeled and manufactured with 3D printing.

Several other computational tools for automated and tileable microstructure design have been recently introduced, such as a framework for two-scale topology optimization^28^, tileable microstructures from families of related structures^29^, tileable microstructures derived from Voronoi diagrams induced by star-shaped metrics^30^, 3D star-shaped tile sets emerging from a discrete growth process in a lattice^31^, computationally mapping auxetic, conventional, and transitional unit cells into cellular solids^32^, and using combinatorial search over topologies to obtain parametric cubic patterns with isotropic elastic materials^33^. These methods have generated fascinating metamaterials with applications in topology optimization^28^, controlled elasticity^29,33^, shape-matching^32^, and others. Our method builds on key ideas from these and possesses alternative benefits. For example, rather than pre-computing unit cells with combinatorial search^28,33^, or pre-defined “families”^29,32,34^, using StyleGAN as an automated generator operating on a continuous latent space enables on-the-fly generation of various smoothly transitioning unit cells. This further enables gradient stacking in the z-direction for complex three-dimensional structures. Additionally, using an algorithm to generate structures inspired by natural materials may capture statistical variations or other nuances that are favorable for specific applications, such as by enhancing delocalized buckling. Mathematical abstractions or human design, alternatively, can sometimes be critical engineering tools but may not capture such nuances^35–37^.

Here, we demonstrate these concepts end-to-end by training StyleGAN on a dataset of leaf micrographs and generating de novo architected leaf-inspired materials in several different ways. Leaves represent interesting natural materials that, while hierarchically structured, are typically bound to 2D geometries by evolutionary limitations such as transpiration processes^38^. Using StyleGAN as a platform, we explore the extrapolation of leaf geometries into three dimensions, and demonstrate several tools for generating, designing, and optimizing architected nature-inspired materials.

## Results and discussion

### Leaf-inspired material unit cells

Training the StyleGAN architecture on leaf micrographs such as those shown in Fig. 1a constructed a 512-dimensional latent space in which every point corresponds to a leaf image. Novel images were accessed through seed values, which were used to generate random 512 × 1 vectors to initiate the trained generator network. Binary processing, island removal, and XY-mirroring of individual leaf images with OpenCV produced symmetric leaf-inspired material “unit cells” that emulate the structure of two-dimensional open-celled foams. Figure 1a depicts this process (more detail is shown in Supplementary Fig. 1) and shows several examples of generated leaf images, along with their corresponding unit cells. Some images display only the midrib and secondary veins of a leaf, while others also display tertiary and/or quaternary veins, and their corresponding unit cells reflect these varying levels of hierarchy in their microstructures. This indicates that the model is able to simulate hierarchical properties present in the training data and shows, at a coarser scale, the model’s ability to generate continuously varying structures.

A library of 1000 randomly seeded leaf images and their corresponding unit cells was generated from the trained StyleGAN model. These unit cells could be visually grouped into three major families (Fig. 1b), which each contained numerous images with slight variations and multiple levels of hierarchy. Additional sample microstructures can be found in Supplementary Fig. 2. Generally, more detailed leaf images yielded more densely architected unit cells, enabling the creation of distinctly hierarchical architectures. Figure 1c shows the density distribution of the unit cell library. While a bimodal distribution is apparent, there are entries that span nearly the full spectrum of densities between 0–1. Material architectures can therefore be generated at any desired density, which is especially important because the relative density (ρ/ρ_s_) of cellular materials largely dictates their mechanical properties as described by the relationship:

1 $$\frac{E}{{E}_{{{{{{{{\rm{s}}}}}}}}}}=C{\left(\frac{\rho }{{\rho }_{s}}\right)}^{2}$$

where E is the effective modulus of the foam, E_s_ is Young’s modulus of the cell wall material, ρ is foam density, and ρ_s_ is cell wall material density^39^.

**Fig. 2:**
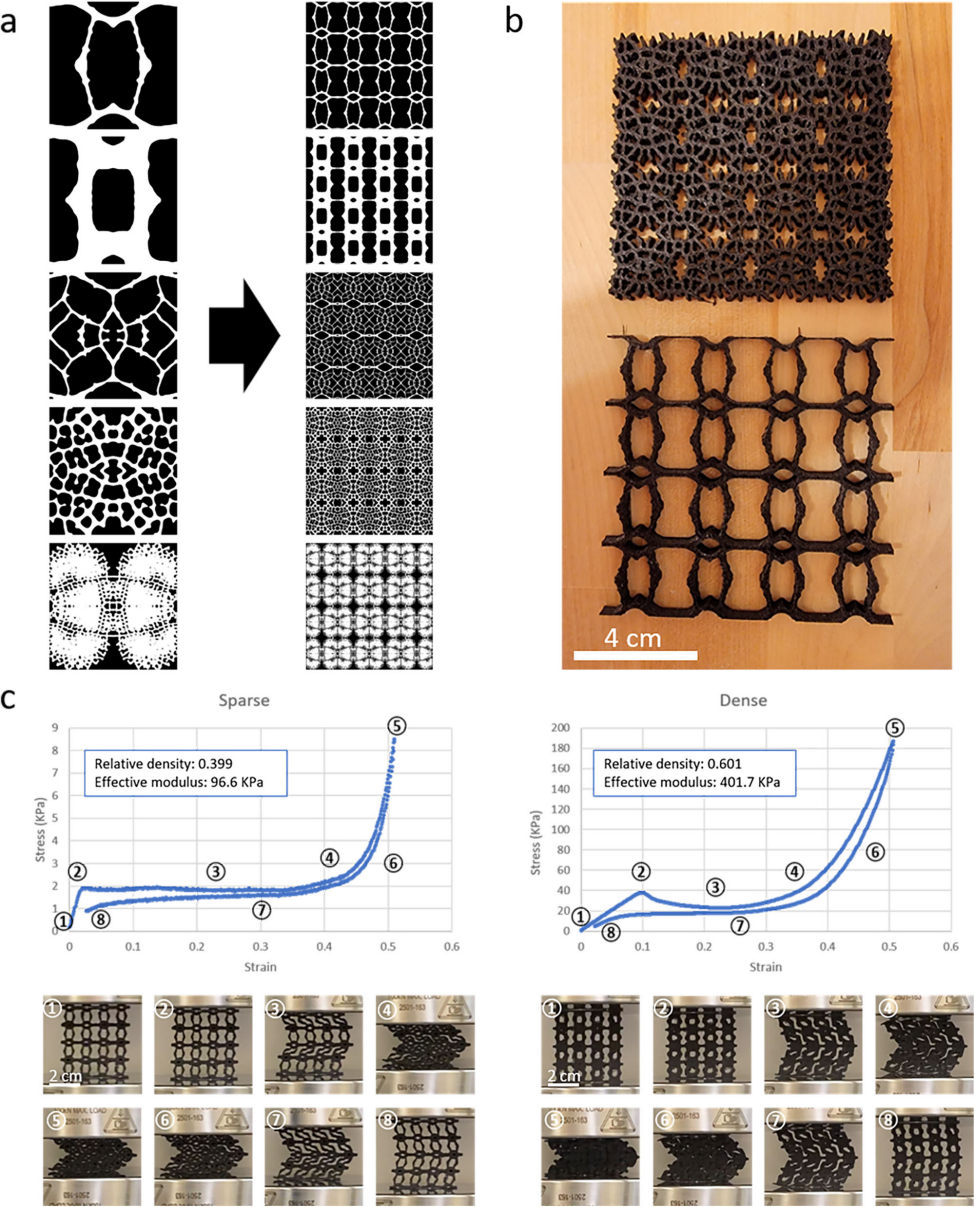
Unit cell tiling into quasi-2D materials with properties of open-celled foams. **a** Symmetric leaf-inspired unit cells can be tiled in both X and Y directions to generate large-area quasi-2D materials. **b** 3D-printed samples of 2D leaf-inspired architectures, one with a gradient of wall-thickness. **c** Simple deformation analysis of two leaf-inspired structures, one with relatively low density and one with relatively high density. Both exhibit mechanical properties of elastic open-celled foams, with linear elastic wall buckling between points 1 and 2, a plateau of deformation at near-constant stress corresponding to buckling of cell walls between points 2 and 4, and a sharp increase in stress as cell wall materials are crushed together between points 4 and 5. Because these samples were printed with an elastic material, the structures are largely recovered as strain returns to zero.

From here, the various leaf-inspired unit cells serve as building blocks for crafting a multitude of architected materials. At its simplest, unit cells can be tiled in X and Y directions to produce large-area two-dimensional architected materials. The mirroring operation performed in generating unit cells guarantees that they are symmetrical and tileable, including between closely related units, to enable the creation of gradient structures. Figure 2a depicts several examples of tiled “leaf-inspired” unit cells, and Fig. 2b depicts physical 3D-printed samples. The cell wall thickness of individual unit cells can also be varied in post-processing to generate graded 2D materials.

The mechanical analysis confirmed that the tiled 2D architectures behave like open-celled foams, as expected^39,40^. Figure 2c shows the stress-strain curves of two handpicked 2D architectures tiled from seed values 441 and 863, which had similar architectures but lower and higher relative densities, respectively, 3D-printed in an elastic material. Both curves show three distinct regions; a linear elastic region corresponding to the elastic bending of cell walls, a plateau of deformation at near-constant stress corresponding to the buckling of cell walls, and a sharp increase in stress as cell wall materials is crushed together.

### Image manipulation

The infinite images that can be generated from the latent space serves as an expansive and continuous design space from which to produce leaf-inspired architectures. While this is indeed beneficial, for specific applications, methods to search or manipulate the design space to find architectures with desirable properties are necessary. Two such methods, image projection/encoding and image mixing are described below.

**Fig. 3:**
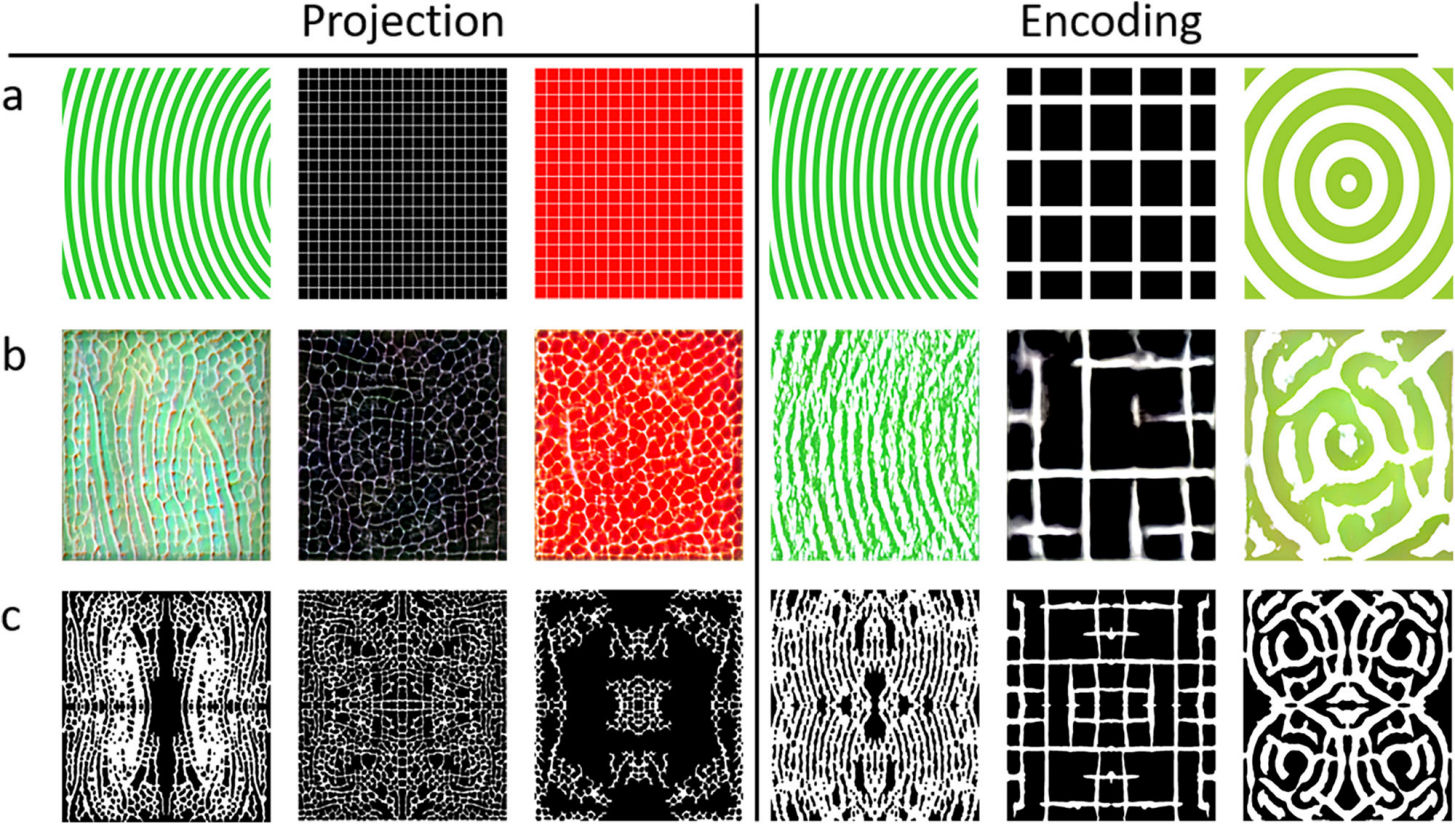
Image projection and encoding to guide the generation of leaf-like structures. Examples of using image projection and encoding to guide the generation of leaf-like structures. The top row (**a**) depicts the projected target images, and the middle row (**b**) depicts images generated by the model from the matching latent vectors found. The bottom row (**c**) shows processed and mirrored tileable unit cells produced from the generated images in a row (**b**).

Image projection involves designating a target image and backpropagating through the generator model to find a latent vector *w* that most closely matches it. Target images are projected onto the latent space by optimizing *w* over many iterations while penalizing a loss function that quantifies the distance between the target image and the synthesized image^27^. Thus, any image can be generated in a “leaf style” with the trained model, as shown in Fig. 3. This allows manual input of design cues for generated images, and, therefore material architectures, which is advantageous when specific forms, geometries, or hierarchical levels are required. We further distinguish between the nuances of projection and encoding: As previously discussed, the latent *w-*vector is input into multiple layers of the generator, which contributes to feature separation and multi-level control (generator architecture can be found in Fig. 1a). Image projection identifies a single *w-*vector common to all input layers of the generator, whereas encoding, an extension of this, allows a different *w*-vector to be optimized for each layer. Projection retains more characteristic features of the generator (i.e., what makes a leaf a leaf), while the encoding allows greater visual fidelity by enabling the generation of images that do not have a “pure” latent representation. As a result, projection sometimes cannot emulate images that deviate too much from leaf microstructures, while encoding can replicate target images with higher fidelity at the cost of losing the nuance of leaf microstructures, although this can be curbed by stopping the optimization early.

Both these methods are valid tools depending on application goals. Several projected and encoded images are shown in Fig. 3, along with their corresponding target and processed “unit cells”. As evident, projected images have more characteristic leaf-microstructure-like cells, whereas the encoded images are able to emulate the target images more closely with some leaf-like properties. Projecting and encoding appear to be sensitive to a number of parameters, including shape and color. Fascinatingly, the model can generate images in black and red, colors which did not appear in the training data. While color is not especially important in binary material architecture design, this implicates the expanse of the latent space and the generative abilities of the model beyond the properties of the existing data.

Generating guided structures with leaf “styling” can be beneficial in engineering applications. For example, natural variability from leaf structures contributes randomness to architected materials, which can foster delocalized buckling and higher energy dissipation for failure-resistant structures^41^. Structural randomness is present in nearly all cellular materials in nature, including bone and nacre^42^.

**Fig. 4:**
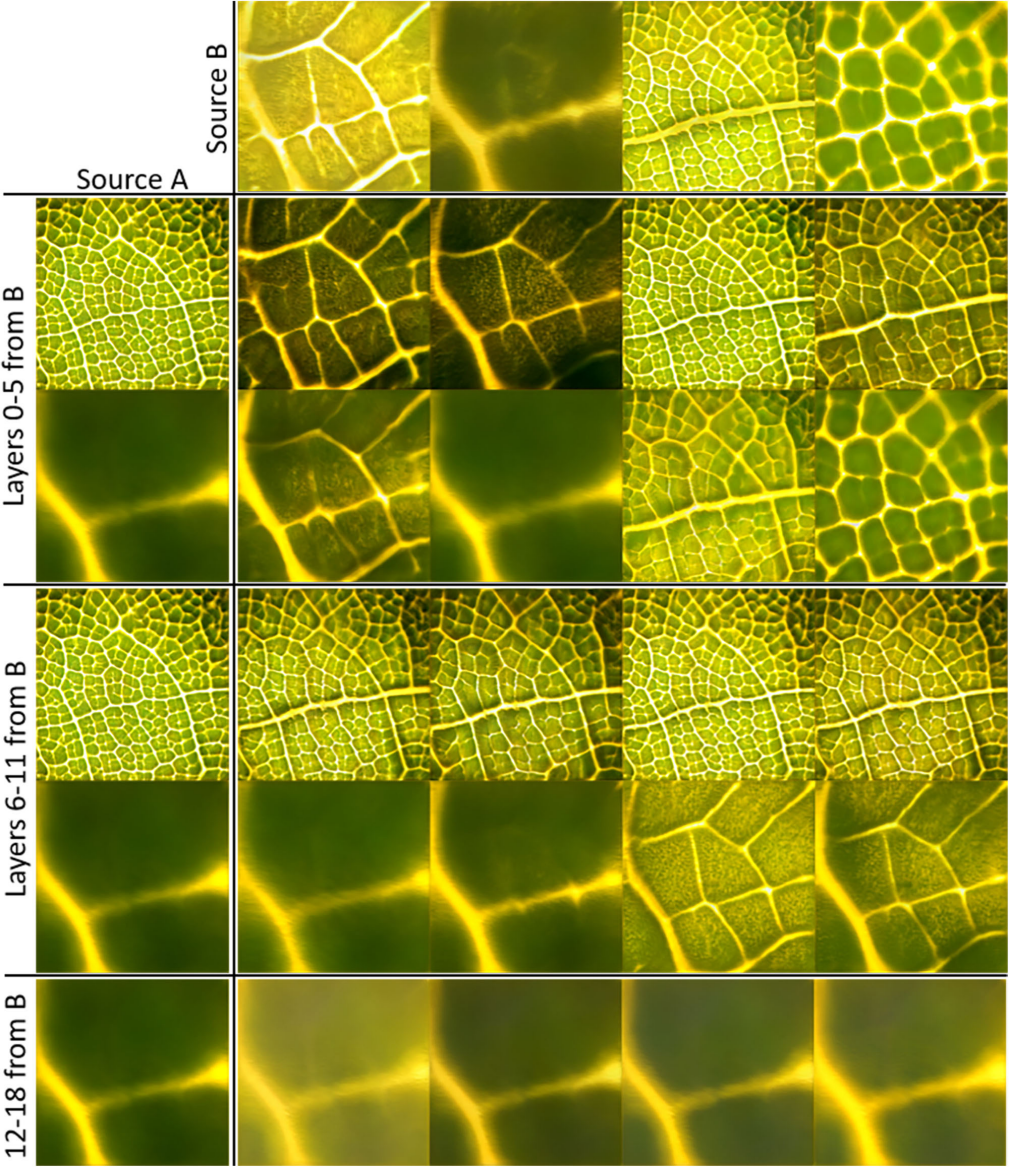
Style-mixing of generated leaf images. Examples demonstrating the style-mixing method. Using the latent code for Source B in earlier layers of the generator causes heavy influence from B on the image’s higher-level features, such as structure. Using latent code for Source B in downstream layers of the generator influences finer features such as color scheme.

Leaf images can also be generated by combining desirable qualities from multiple different images. Termed “style-mixing” ^24^, this is accomplished by generating an image with multiple *w*-vectors specified by seed values, similar to the encoding process described above. Using the latent code for one “pure” image in earlier layers of the generator corresponds to copying the image’s coarse spatial resolutions, and, therefore, high-level aspects such as the main structure. Downstream layers of the generator correspond to finer features such as color schemes. As such, by carefully selecting which latent codes are input to each layer in the generator, a leaf image can be crafted such that it contains designated features and qualities, and can be converted into a desired leaf-like architecture. Style-mixing is demonstrated in Fig. 4. In particular, note how images largely take on the architecture from Source B when the *w-*vector for layers 0-5 is taken from B, while the images take only color from Source B when the last layers of the generator use the B *w-*vector. Taking middle layers from B somewhat mixes both color and hierarchical complexity from sources A and B. With a more comprehensive training dataset, the model may be trained to control features that are more relevant to material architecture than color, such as wall thickness. Style-mixing expands the design space delineated by the model’s latent space, and further enables directed design through fine-tuning of generated images or even projected images already engineered to have specific qualities.

### 2D materials by design

Not only can leaf-inspired unit cells be tiled in X and Y directions to create large-area materials, the StyleGAN latent space can also be used to generate materials with varying structures and properties. Because two points close to each other in latent space correspond to similar images, small steps in latent space create smooth interpolations between features, which in the case of material architectures, generates smoothly evolving structures. With small enough steps, enough similarity is maintained from one generated structure to the next that these can also be tiled next to each other while maintaining continuity. Different movements in latent space output different material architectures.

Various movements in latent space can be explored by defining a reduced-dimension coordinate system spanned by three points in latent space with:

2 $${{{{{{{\bf{x}}}}}}}}=({{{{{{{\bf{b}}}}}}}}-{{{{{{{\bf{a}}}}}}}})$$

3 $${{{{{{{\bf{y}}}}}}}}=({{{{{{{\bf{c}}}}}}}}-{{{{{{{\bf{a}}}}}}}})$$

where ${{{{{{{\bf{x}}}}}}}}$ and ${{{{{{{\bf{y}}}}}}}}$ are the vectors that define the x- and y-axes, respectively, and ${{{{{{{\bf{a}}}}}}}}$, ${{{{{{{\bf{b}}}}}}}}$, and ${{{{{{{\bf{c}}}}}}}}$ are the latent vectors defining three selected points, each of which corresponds to a different leaf image and generated architecture. Then, points in the coordinate system can be accessed with:

4 $${{{{{{{\bf{d}}}}}}}}={{{{{{{\bf{a}}}}}}}}+x* {{{{{{{\bf{x}}}}}}}}+y* {{{{{{{\bf{y}}}}}}}}$$

where ${{{{{{{\bf{d}}}}}}}}$ is the vector defining a new point to be generated, and *x* and *y* are multipliers between 0 and 1.

**Fig. 5:**
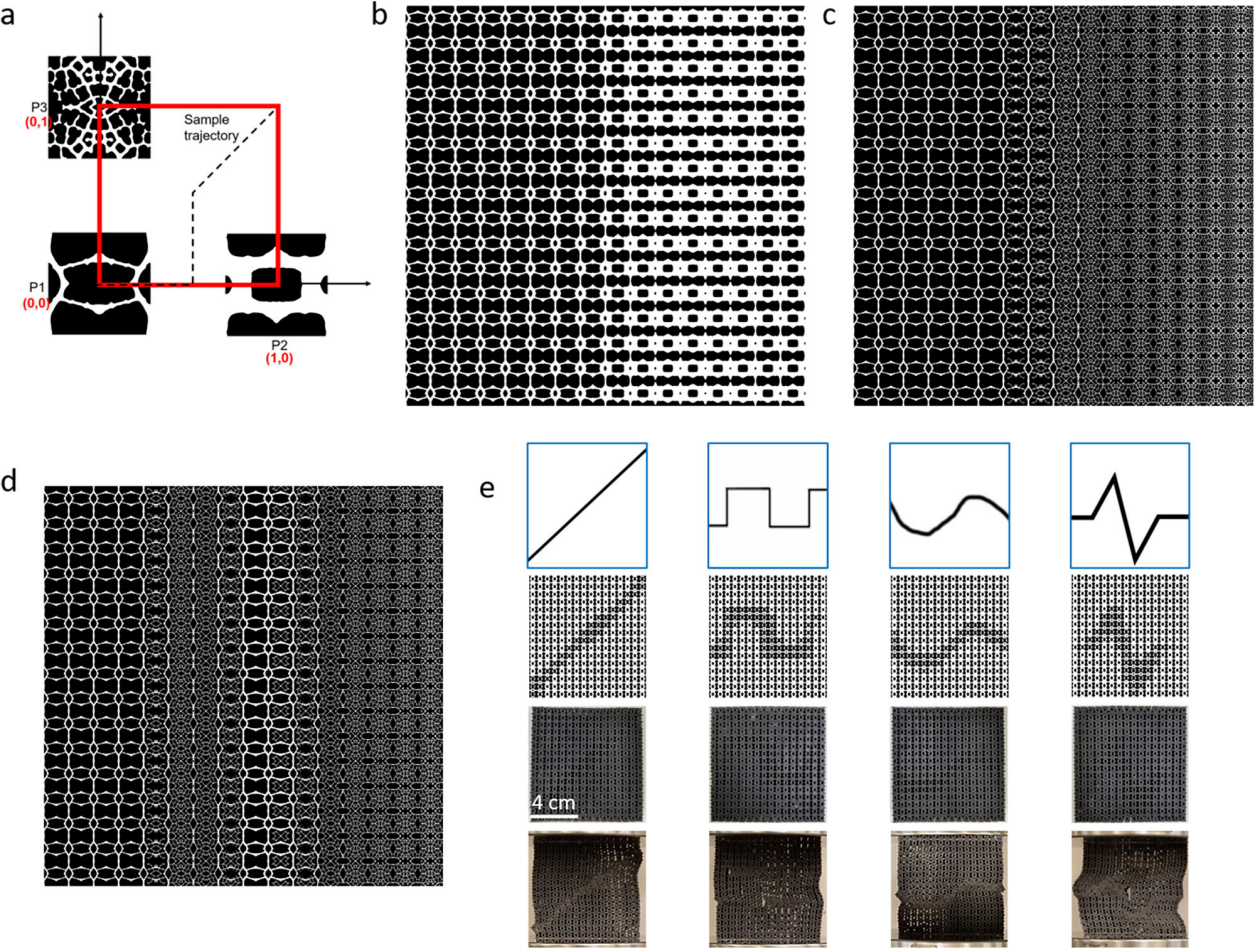
Reduced-dimension coordinate systems in latent space to generate gradient or smoothly varying structures. **a** Example of a reduced-dimension coordinate system, spanned by three points in latent space (P1, P2, and P3). **b**, **c** Continuously evolving gradient structures generated with small steps through the reduced-dimension coordinate system from P1 to P2 (**b**) and from P1 to P3 (**c**). **d** Continuous structure generated by following the segmented sample trajectory shown in (**a**). **e** Samples generated with the Img2Architecture tool, including the source images (top), the generated architectures (second row), the 3D-printed architectures (third row), and the printed architectures under compressive loading (bottom), demonstrating designed buckling.

An example is shown in Fig. 5a. Traversing across this coordinate plane is equivalent to interpolating between vectors **a, b**, and **c**, thus generating images that are various combinations of their corresponding images. For example, the architecture depicted in Fig. 5b was generated by traversing across the “x-axis” from (0,0) to (1,0) on the coordinate system defined in Fig. 5a. Similarly, Fig. 5c depicts the latent walk from (0,0) to (0,1). As evident, the architectures smoothly evolve from one unit cell to another. With specified unit cell geometries, this presents a method for creating materials with gradient microstructures. This concept applies to any walk-in latent space in the designated coordinate plane. Figure 5d shows the structure generated from the sample trajectory delineated in Fig. 5a. The leaf image slices output in this trajectory are shown in Supplementary Movie 1 to demonstrate their smooth and continuous transitions. Other deliberate designs can be projected into latent space using this coordinate system, such as circular motions for periodic designs. In this way, not only can StyleGAN be used to construct novel materials that mimic natural designs, it also allows for a transfer of information across manifestations, using the latent space as the mediator.

In Fig. 5e, we extend this concept and demonstrate Img2Architecture, a tool to create materials by design using images as stencils to generate architected materials with designated property distributions. Further details of the process can be found in Supplementary Fig. 3. The examples shown use the thin and thick-walled unit cells previously discussed to demonstrate material architectures that exhibit patterned buckling. The lower-density unit cells have a lower effective yield point than the high-density architectures, as shown with mechanical testing, thus are expected to buckle first under loading^39^. As such, materials with controlled bucking are created by converting images that designate where buckling is desired into architectures with lower density in the buckling zones. Unit cells surrounding the pattern are interpolated with a gaussian process to ensure continuity and smooth transitions, enabled by continuous variation generated through small steps in latent space. Figure 5e depicts the image sources, the resulting architectures, the 3D-printed architectures, and the printed samples under approximately 15% strain. Note how the material architectures deformed where patterned, however, as especially evident in the rightmost pattern, the connectivity between unit cells was not always perfect. Nevertheless, with further optimization of the model and method, this concept may see a potential application in soft robotics with planned and reversible deformation, mechanical filtration with reversibly changing pore sizes, and other applications that require multifunctionality. Simply by selecting different points in latent space that correspond to unit cells with desirable properties, this tool can easily be applied to using images as stencils for architected materials with various applications.

### Movements in latent space: 3D architectures

**Fig. 6:**
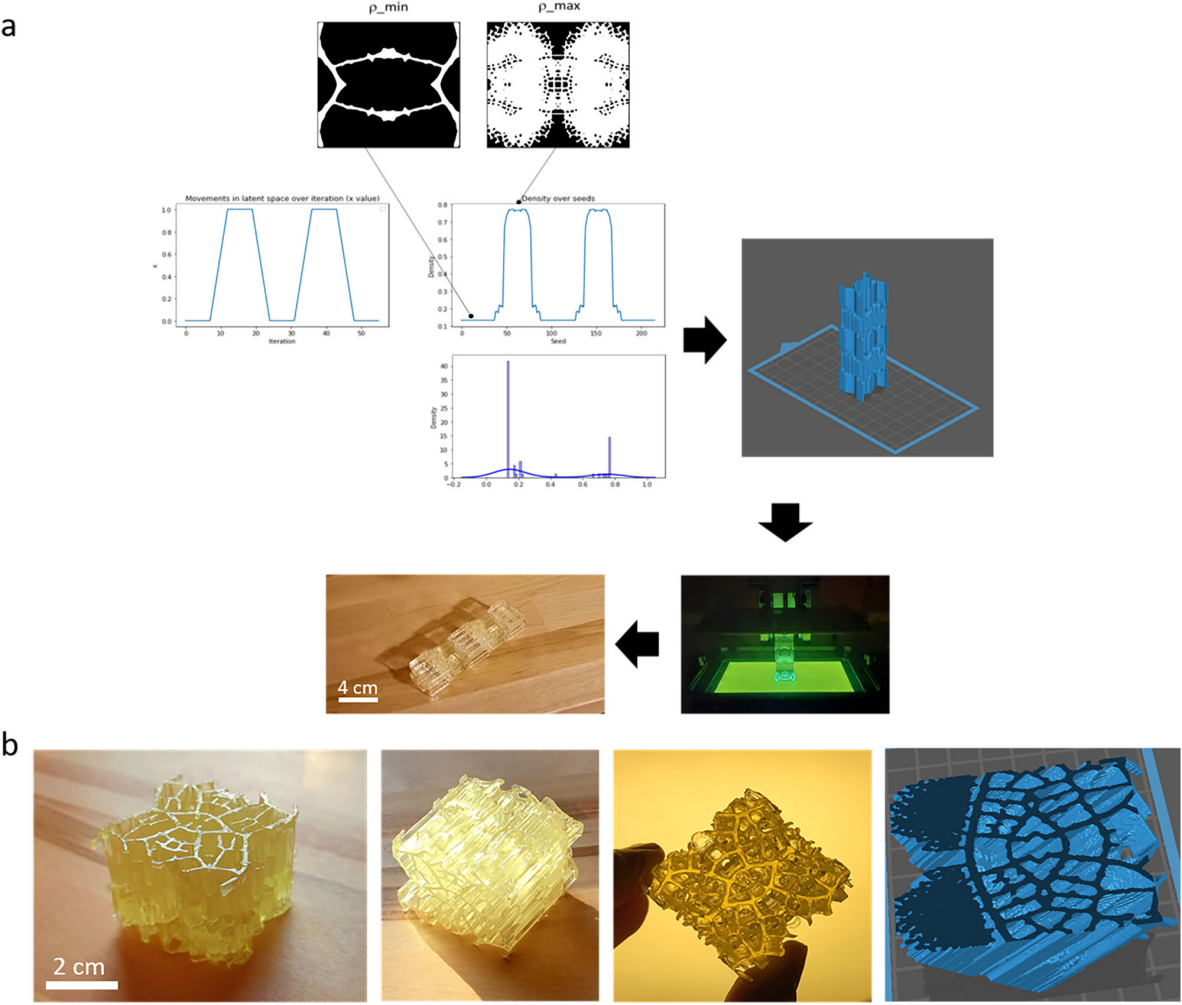
Inverse tomography stacking into 3D architectures. **a** Using designed movements in latent space, desired 3D materials can be constructed. Here the latent space is mined for the highest and lowest density microstructure based on a set of 200 random seeds, and then interpolation between the two extreme density regions is used to construct a periodically varying architected material that uses these two building blocks as design elements. The resulting design is 3D-printed. **b** A 3D model generated by smoothly interpolating between the three major architectures identified in generated images, as shown in Fig. 1. Shown are a 3D-printed model, and a diagonal cross-section of the digital model to demonstrate connectivity.

With even smaller steps in latent space, continuous slices of output images can also be stacked into 3D structures in an inverse tomography approach. Thus, unit cell architectures can be smoothly varied as they are stacked in all three X, Y, and Z directions to create materials engineered to have varying properties. Modern advances in additive manufacturing technology then enables the manufacturing of these complex forms. Figure 6a shows one example where the highest and lowest density microstructures in the unit cell library were used to construct a periodically varying architected material that interpolates between these two extreme density regions. Figure 6b shows a material generated by smoothly interpolating between two of the major microstructures previously shown in Fig. 1, as well as a diagonal slice of the digital render to show internal connectivity. For further detail, an animation of the slicing through the entire geometry is shown in Supplementary Movie 2. As evident, this interpolation enables the generation of a complex open-celled material that emulates leaf microstructures in three dimensions. When creating a gradient from one microstructure to another, the number of steps taken between the microstructures dictates how smooth the transition is. The 3D structure in Fig. 6a, for example, took only six transitional steps in the latent space before repeating the extreme density microstructures, while the example in 6b was constructed entirely of the linear interpolations between microstructures over nearly 250 steps each.

In future work, by combining tiling techniques in X, Y, and Z directions with simple mapping operations, these methods can be used to create large-scale geometries that vary in all three directions. Furthermore, with the appropriate training dataset, the generative model may be able to achieve enough structural variety and resolution in continuous change to generate structures that stack into closed-cell foams.

### Structure simulations, evaluation, and optimization

**Fig. 7:**
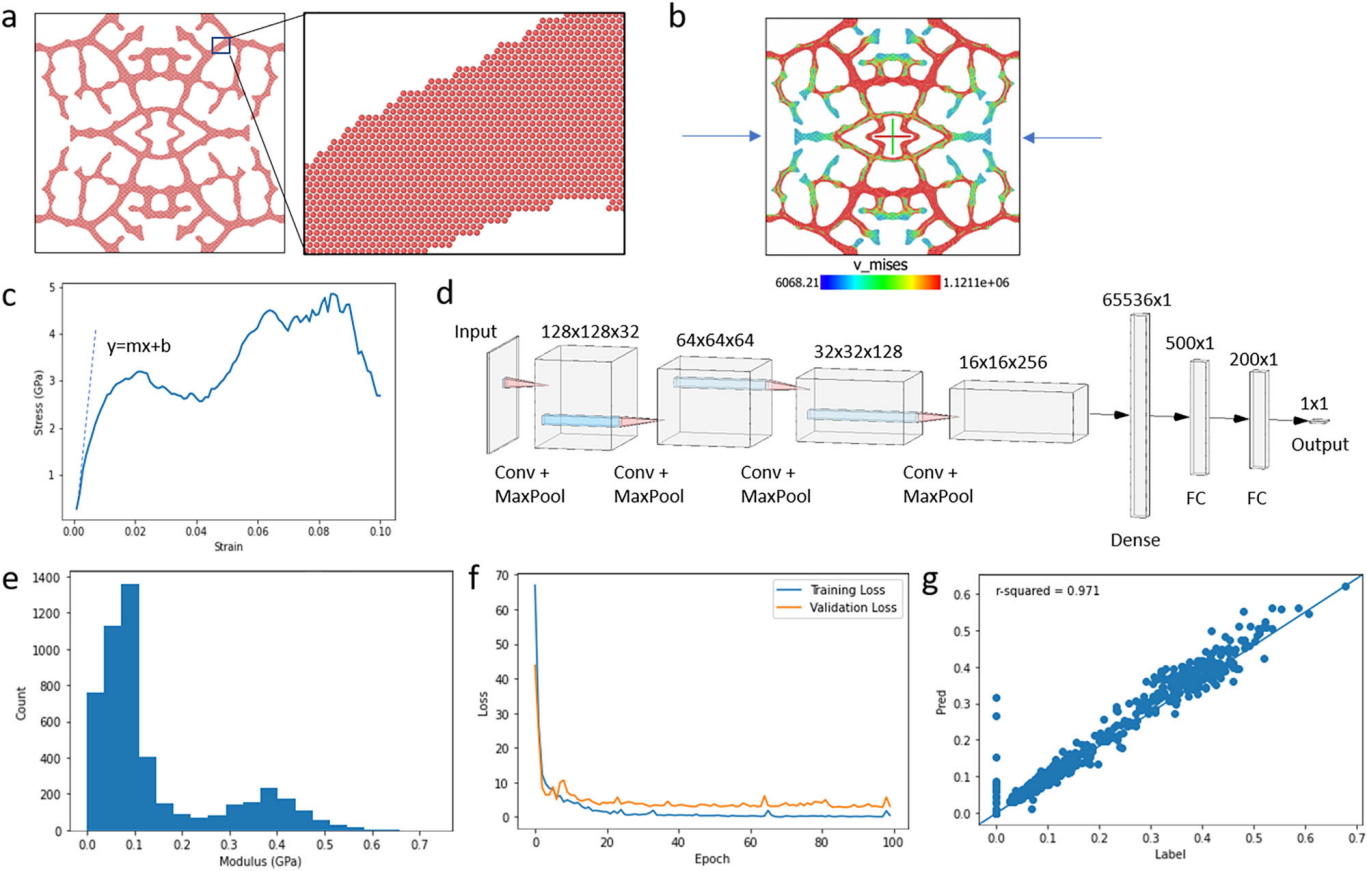
Compression simulations and subsequent CNN training. **a** Coarse-grained “atomistic model” of a StyleGAN generated structure for simulation in LAMMPS. **b** Coarse-grained model under uniaxial compression in the x-direction. Colormap shows Von Mises stress. **c** Stress and strain experienced by the model in (**a**) during simulated uniaxial compression, along with a demonstration of using the linear elastic region of the stress-strain curve to determine the effective modulus. Here, the effective modulus is equal to the slope of the linear fit. **d** Four-layer convolutional neural network used as a surrogate model to predict the effective modulus of a structure from its image. **e** Distribution of the normalized data (normalized with the effective modulus of a solid structure) used to train the convolutional neural network (CNN). **f** Training curve of the CNN; note that labels were scaled by 100x for model training, resulting in the large loss values shown. **g** CNN predictions on the test set plotted against corresponding labels to demonstrate CNN performance.

Compression simulations with uniaxial deformation were used to acquire high-throughput mechanical information on generated structures. In short, 2D coarse-grained models were constructed for each structure by tiling a hexagonal unit cell of “atoms” per pixel of material in the image of each structure, shown in Fig. 7a, and these models were simulated under uniaxial compression with 2D periodic boundary conditions and Lennard-Jones potentials (Fig. 7b). Coarse-grained models were chosen to simulate mechanical loading because finite element analysis incurred a higher computational load for our complex geometries. The simulations generated stress-strain curves for each structure, such as the example shown in Fig. 7c, from which effective modulus was calculated. Using such simulations, a large dataset containing 5000 randomly seeded structures was generated, including density and effective modulus for each structure. The effective moduli were normalized by dividing by the modulus of a solid square of material simulated in the same way, and any structures considered invalid due to lack of connectivity were labeled with a modulus of 0. The data distribution is shown in Fig. 7e.

This dataset was split into training (70%), validation (10%), and testing (20%) sets and used to train a surrogate model to predict effective modulus from the image of a structure. The surrogate model, a four-layer convolutional neural network (CNN) shown in Fig. 7d, was trained over 100 epochs and achieved an R^2^ score of 0.971 on the test set. The CNN’s training curve, displayed in Fig. 7f alongside the model’s predictions on the test set (Fig. 7g), shows that the model overfits slightly, but trained quickly and generally performs well.

**Fig. 8:**
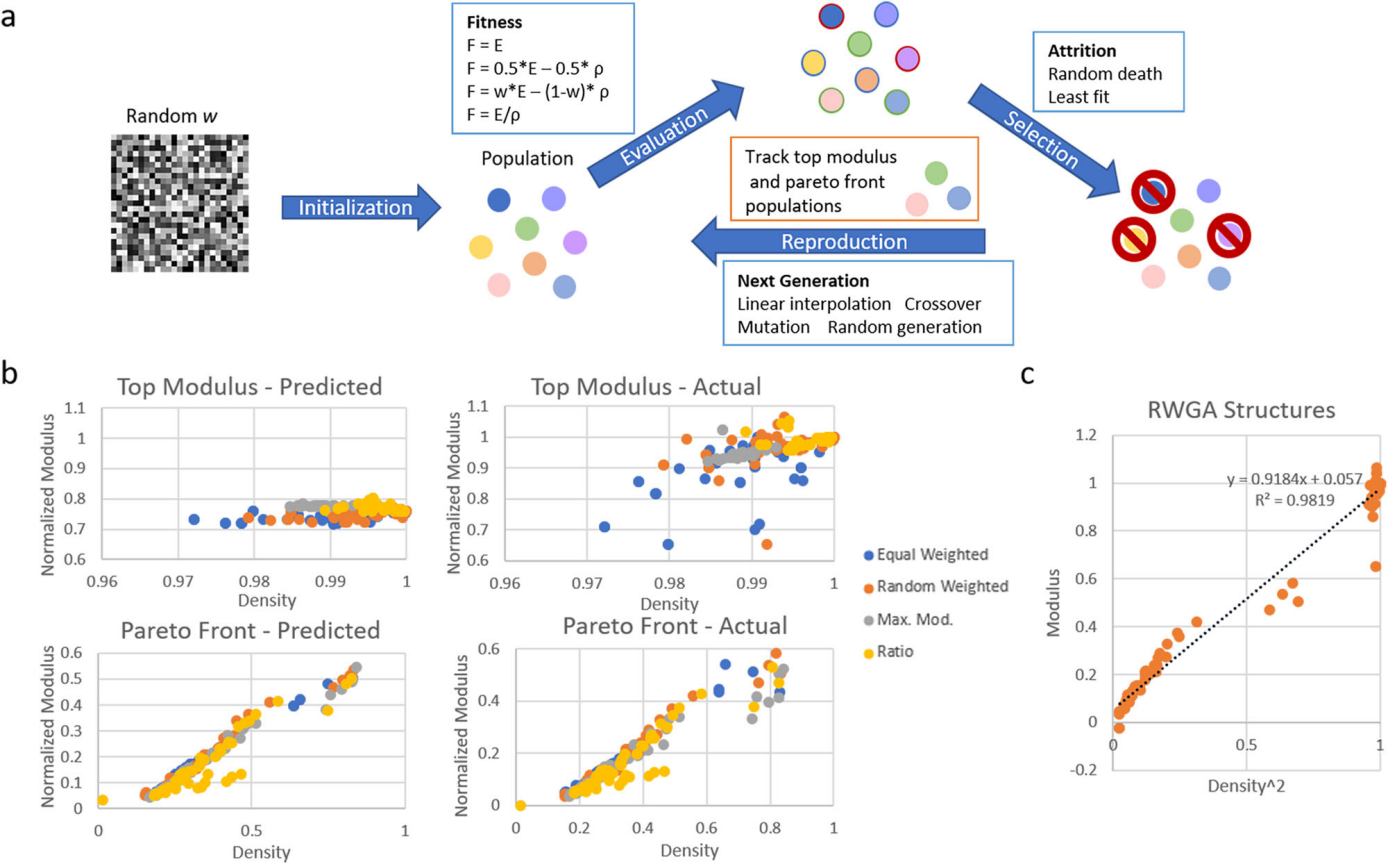
Structure optimization with genetic algorithms. **a** Genetic algorithm (GA) used to optimize structures generated by the trained StyleGAN model. **b** Density and normalized modulus of the 50 individuals predicted to have the highest modulus and the 50 individuals predicted to be the most Pareto efficient for each of the four fitness functions used in the genetic algorithm. Also shown is the actual density and modulus of these individuals as determined by coarse-grained simulations of uniaxial compression. **c** Density-squared plotted against modulus for the top modulus and Pareto front populations found by random-weighted GA. The high r-squared value of the linear fit (dotted blue line) demonstrates that this population follows the expected behavior of cellular materials described in Eq. 1.

Finally, a genetic algorithm (GA), summarized in Fig. 8a, was used to optimize structures generated by the StyleGAN model based on effective modulus. The GA used the CNN surrogate model as an evaluator rather than embedding simulations directly to enhance the speed of the GA and decrease computational cost. For reference, the trained CNN could predict the modulus of an individual structure in less than 1 s, while running a simulation took ~3 min. The GA uniquely operated directly on latent code from StyleGAN’s intermediate latent space *w*, using *w*-vectors as the “genetic code” to take full advantage of the plasticity of the StyleGAN generator. Because a *w*-vector, typically 1 × 512, is input into the generator at 18 different layers, each “individual” in the GA’s population was a structure defined by 18 512 × 1 vectors, or “genes”. This allowed for different vectors to be used in each layer of the generator, similar to the image encoding and style-mixing methods previously described.

The GA was initialized by randomly seeding a population of 10 “pure” individuals whose genetic codes each contained 18 identical “genes”, meaning that these were each individuals the StyleGAN generator could produce from a single seed value. Then, in each generation, “parents” were selected from the population with probabilities based on individual fitness, and “children” were created from them in three ways: linearly interpolating between the vectors in each parent’s gene code, performing gene crossover by randomly choosing which parent to take each of 18 genes from, and imposing point mutations by slightly changing individual vector entries based on pre-defined gaussian distributions. New “pure” individuals were also introduced in each generation with random seeding. A total of 7 new individuals were added to the population in each generation, after which three random individuals and the four least fit individuals were removed to maintain a population size of 10. This large population turnover was designed to emphasize the exploration of the StyleGAN latent space as compared to exploitation-focused strategies.

Four different evaluation schemes were investigated for the GA with two goals: optimizing structures for maximum modulus, and maximizing modulus while minimizing density to find the optimal Pareto front. As such, each time the GA was run with a different evaluation scheme, 50 individuals with the highest modulus and 50 most Pareto-efficient individuals found by the GA were separately tracked. The first fitness function equated the fitness of each individual to its effective modulus as predicted by the CNN, directing the genetic algorithm to find the highest modulus individuals within the expanded latent space allowing for mixed genes. To better balance modulus and density, fitness schemes that equally weighted modulus and density in a single optimization function, that randomly weighted modulus and density each time an individual was evaluated, and that optimized for the ratio between modulus and density were investigated.

The “top modulus” and “Pareto front” populations for each evaluation scheme after 1000 generations are plotted in Fig. 8b, including each individual’s CNN-predicted modulus and actual modulus as found through simulations for validation. Interestingly, the surrogate model generally under-predicted the modulus of “top modulus” individuals. This may be because the dataset it was trained on did not include individuals with such high modulus—in fact, the highest modulus structure in the training dataset had a normalized modulus of 0.732 with a density of 0.912. Incredibly, nearly every individual in the “top modulus” population exceeds this modulus, albeit with higher density. Two of the structures even achieved moduli higher than that of a solid structure, which may be an artifact of the automated modulus calculation algorithm rather than reality, but is highly indicative of successful modulus optimization. The surrogate model predicted the moduli of the “Pareto front” populations quite well.

**Fig. 9:**
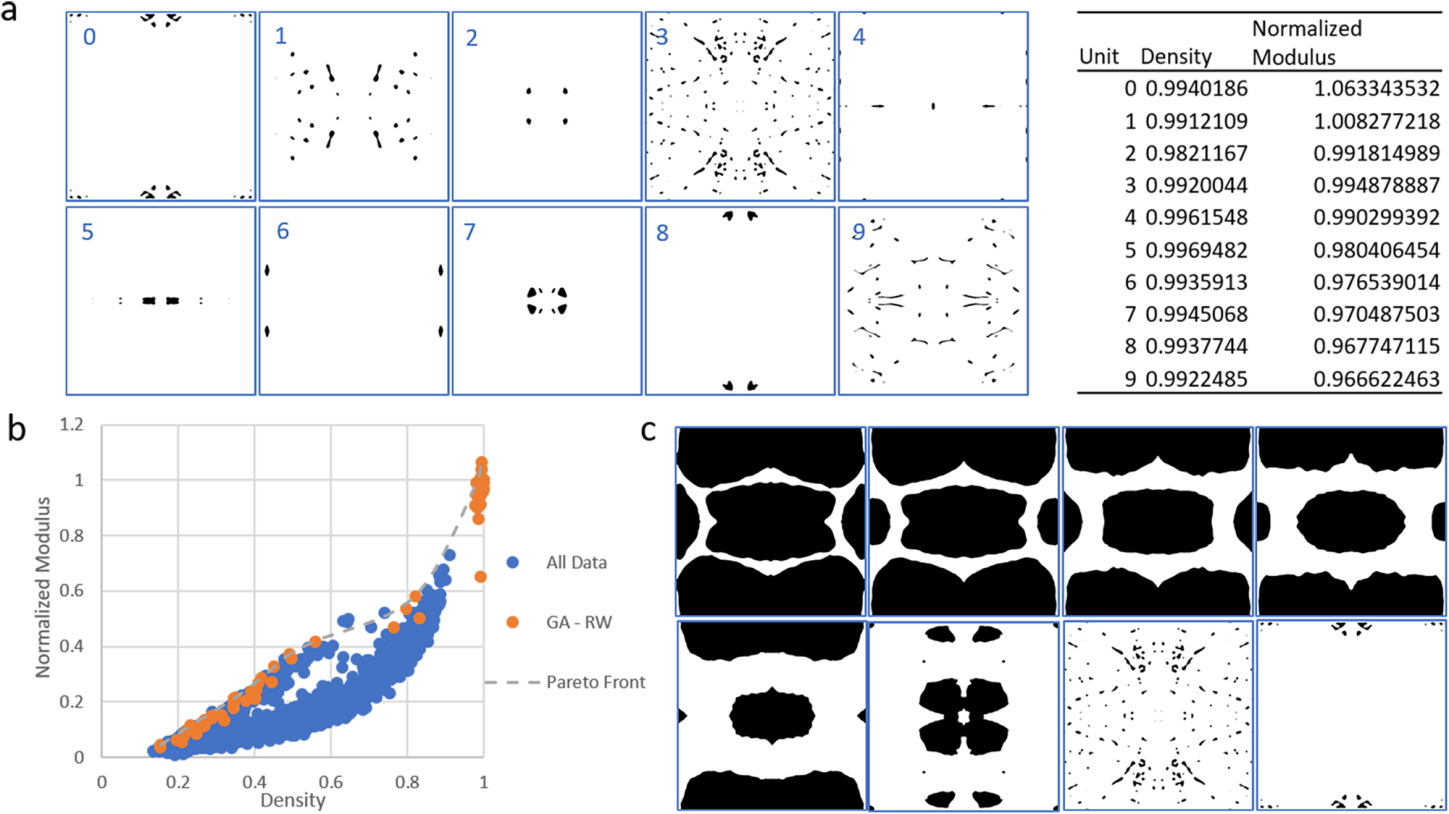
Random-weighted genetic algorithm results. **a** Ten top modulus individuals were found by the random-weighted genetic algorithm (RWGA), along with their density and normalized modulus as determined by atomistic simulations of uniaxial compression. **b** Top 50 Pareto-efficient solutions found by the RGWA compared to the density-modulus distribution of the training data. **c** Eight Pareto-efficient solutions of varying density found by the RGWA.

Density-squared versus simulated modulus from both “top modulus” and “Pareto front” populations of the random-weighted GA, which appeared to have especially good performance based on visual inspection, are shown in Fig. 8c to show alignment with the expected behavior of cellular materials described by Eq. 1. Figure 9 displays the top ten modulus structures found by the random-weighted GA. Interestingly, when operating on the StyleGAN latent space, the GA was able to extrapolate to higher modulus structures than the StyleGAN model could originally generate, but was generally only able to outline the Pareto front of the training data rather than exceeding it (Fig. 9b). This is further evident upon inspection of the structures that outline the GA Pareto front (Fig. 9c), which closely resemble structures in the training data until moduli higher than those found in the training data are achieved. This may be a result of the limited dataset originally used to train the StyleGAN model, which limits the generative ability of the model. Even heavier emphasis on exploration in the GA or allowing it to operate longer could potentially allow for the discovery of more Pareto-optimal structures. Incorporating the newly generated structures, including high modulus/density structures, into training data for the CNN and StyleGAN could similarly improve performance by enhancing both predictive accuracy and generative ability.

### Incorporation of new structures

**Fig. 10:**
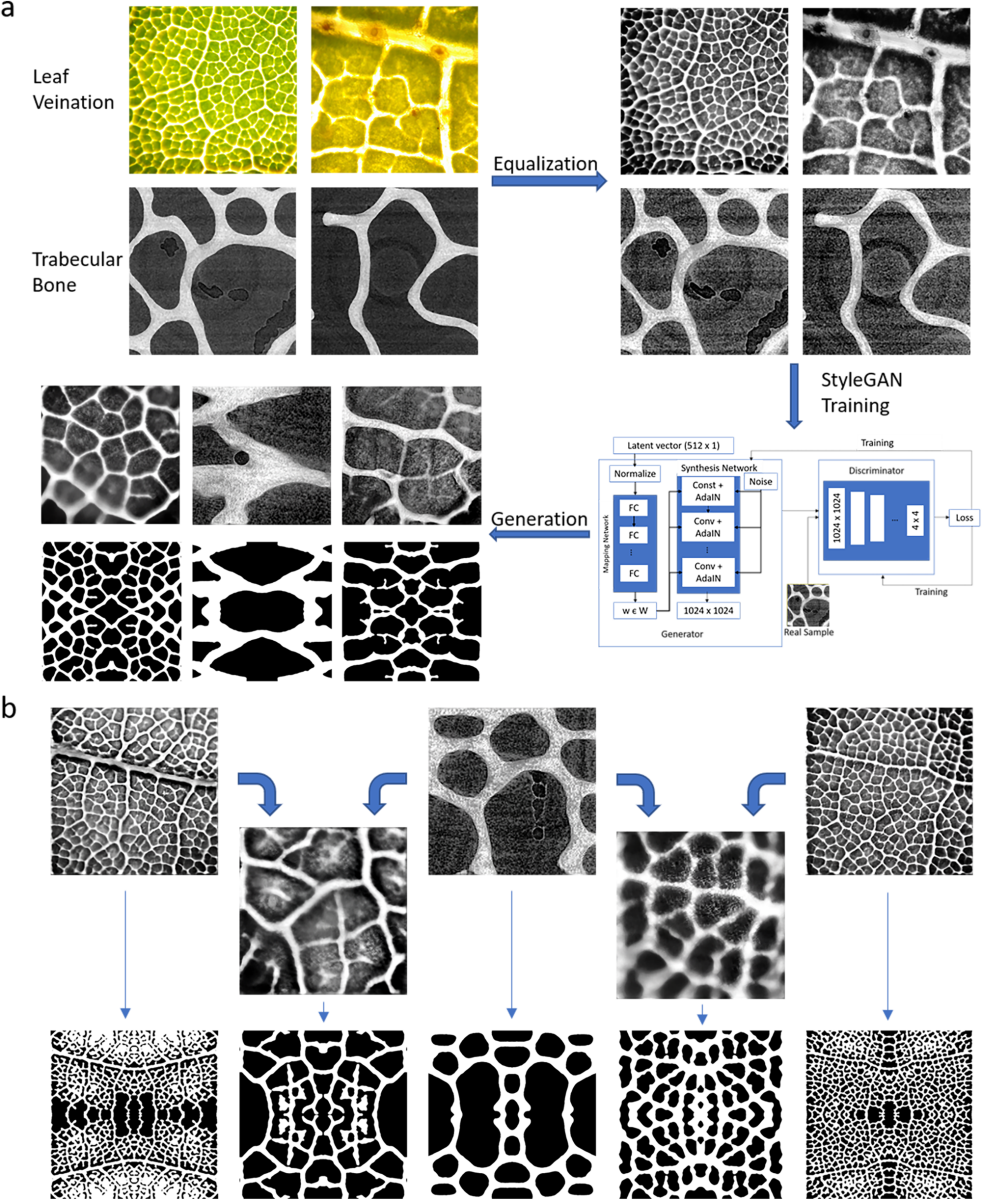
Image generation and manipulation with mixed leaf and trabecular bone. **a** StyleGAN training and image generation with a combination of leaf venation and trabecular bone images. Training images were equalized to have similar grayscale coloring and contrast before training StyleGAN. The trained StyleGAN model was then able to generate images that resemble leaf venation (left), trabecular bone (center), and a combination of the two (right), yielding novel microstructures (below). **b** Style-mixing of leaf-like images with high degrees of hierarchy and bone-like images with only coarse geometry, generating images and corresponding structures with novel architectures and varying degrees of hierarchy.

To explore the interactions of different structures within the StyleGAN latent space, especially with the intent to expand the generative design space, the StyleGAN model was retrained on a combination of the original leaf dataset and an equivalent number of images of trabecular bone. The images were equalized, as shown in Fig. 10a, to discourage the model from focusing on parameters unimportant to structure, such as color. Upon training, the model was able to generate images that resembled leaves, images that resembled trabecular bone structures, and images that appeared to have qualities of both. Critically, these images yielded new microstructures completely different from those generated by the model only trained on the leaf dataset.

Figure 10b demonstrates how images generated by this Bone-Leaf model can also be combined with the style-mixing strategy previously described. Here, it is especially apparent how bone images can take on leaf-like qualities, such as internal cellular structure or vice-versa, such as taking on a more macro-level trabecular geometry. These images, too, yielded novel microstructures. In particular, combining large trabecular geometries with small-celled leaf geometries enabled the generation of unit cells with varying degrees of hierarchical structure.

## Conclusions

Overall, we have demonstrated a method for how the generative StyleGAN algorithm can be harnessed towards utilizing patterns in natural materials for designing engineered materials. Using a leaf dataset, we generated a library of architected unit cells, demonstrated methods for further refining generated structures, and established procedures for using the latent space for the transfer of information across manifestations in the directed design of 2D and 3D materials. We further performed uniaxial compression simulations to gather high-throughput mechanical information on generated unit cells, focusing on effective modulus as a proof-of-concept, and used a genetic algorithm with a CNN-based evaluator to generate optimized unit cells within the StyleGAN framework. Many microstructures were found with moduli that surpassed all microstructures in the training dataset, however, GA-optimized structures were not generally able to surpass the density-modulus Pareto front of the training data. This is likely attributable to the limited leaf dataset StyleGAN was originally trained on, which limits its generative design space. While “gene crossover” enabled the generation of some new structures, the leaf StyleGAN model was generally limited to three main structural families. In contrast, leaves found in nature are infinitely varying. This indicates the need for a larger, more comprehensive training dataset to allow the model access to greater structural variety, in turn expanding its generative abilities and allowing smoother transitions between very different structural forms.

We began to demonstrate this with the incorporation of trabecular bone images, which yielded unique microstructures with varying degrees of hierarchy. Further data with better distributions or higher complexity, including other natural or even synthetic structures, can be incorporated to broaden the model’s design space, which can then be mined using higher-fidelity evaluation methods and similar genetic algorithms.

This method can be applied to other classes of materials, such as auxetic structures, by collecting properties in addition to modulus such as yield strength, toughness, or Poisson’s ratio from simulated stress-strain curves or by prompting simulations to output additional information, as well as by changing the objective function through which the genetic algorithm searches the latent space. For applied materials, this may require simulations to be normalized by or validated with experimental data.

In future work, we anticipate developing structural datasets designed to optimally engage StyleGAN’s generative abilities. We further intend to refine methods to mine the StyleGAN latent space efficiently, especially to explore the limits of the latent space far from the training data, such as by incorporating conditional generation. Finally, we intend to develop tools for multiscale analysis of our materials, including tiled macrostructures and considerations of anisotropy, to enable the automated development and inverse design of functional bio-inspired metamaterials in 2 and 3 dimensions.

## Methods

### Deep neural network: StyleGAN

The generative model was trained with Nvidia’s StyleGAN2 architecture^24,27^. StyleGAN proposes a generator architecture that utilizes style transfer techniques, which aim to customize the style of an image while preserving its content, such as in translating a photograph into a painting. In StyleGAN, a “style” is calculated from each latent vector with a learned affine transformation, and styles are applied in each convolutional layer of the generator. As different convolutional layers control features at different scales, the model automatically learns the separation of high-level features in the data distribution, such as face shape or hairstyle, in generated images of human faces, distinct from stochastically controlled details such as freckle placement. This enables fine control over such style-defining features in generative steps, which in material architectures, can correspond to different levels of hierarchy. Disentanglement of features further allows good interpolation between extremes of a particular feature. StyleGAN operates on a 512-dimensional latent z-space and corresponding 512-dimensional intermediate latent w-space.

### Dataset development

Leaves were chosen as a source of bioinspiration because they represent interesting natural materials that are typically bound to 2D geometries by biological limitations. To generate the dataset for initially training StyleGAN, leaves were collected from the corresponding author’s backyard and imaged with an AmScope optical microscope. The dataset totaled 95 different images with varying magnification, color, and resolution.

### Image generation and image processing

Image generation was primarily performed using code created by Nvidia for StyleGAN with minor modifications. Tools for creating a reduced-dimension coordinate system within the latent space and to traverse through it based on trajectories defined in CSV files were added. Image processing tools for creating unit cells from leaf images were created using OpenCV, an open-source library of computer vision programming functions. Image stacking was performed with NumPy, a library for matrix and other high-level mathematical functions.

Image projection was performed using code created by Nvidia for StyleGAN. Image projection uses gradient descent to optimize the w-vector for the target image. Loss is quantified by using VGG, a deep CNN trained for object recognition^43^, as a pretrained feature extractor on the target and generated images, then calculating the distance between their representations in the high-level feature space. Image encoding functions similarly to projection while optimizing a different w-vector for each of the 18 layers of the StyleGAN generator (thus optimizing a 512 × 18 matrix rather than a 512 × 1 vector).

Img2Architecture functions by normalizing pixels in a grayscale image between 0 and 1, then projecting pixel values onto the vector that spans between two points in latent space such that 0 corresponds to one point, 1 corresponds to the other, and values in between interpolate linearly between the two points (using Eqs. 2 and 4 where y = 0). Structures corresponding to each projected point in latent space are generated, then stacked in a pixel-wise manner such that the image is reconstructed with varying architecture according to the original image.

STL files for 3D printing were generated using trimesh, a Python library for loading and using triangular meshes.

### Additive manufacturing and testing

Leaf-inspired materials for mechanical testing were 3D-printed using a Stratasys Connex3 Objet500 printer in the Stratasys digital material FLX-MK-S50-DM, which has a Shore A hardness value of 50. Other samples were printed using an Ultimaker S3 with TPU (thermoplastic polyurethane) and PLA (polylactic acid) filament (Fig. 2 and Supplementary Fig. 2) and an Anycubic Photon Mono X resin printer (Fig. 8).

Mechanical testing was performed using a 5 kN capacity Instron Universal Testing System. Samples were loaded under compression at 30 mm/min.

### Structure simulations

Images of unit cell structures were translated into atomistic models using python code designed in-house. Uniaxial compression simulations were performed using LAMMPS (large-scale atomic/molecular massively parallel simulator), an open-source molecular dynamics program from Sandia National Laboratories. Each unit cell structure was subjected to minimization and equilibration steps before undergoing uniaxial deformation with a timestep of 0.001 ps and an engineering strain rate of −0.01/ps. Simulations were performed under NVT (canonical ensemble; constant number, volume, and Temperature) conditions with two-dimensional periodic boundaries. For further details, please see the LAMMPS script in supplementary materials.

Each simulation generated a stress-strain curve for the corresponding structure. From the stress-strain curve, the effective modulus of the structure was automatically calculated by fitting a linear regression to points on the curve up to a strain of 0.02, and taking the slope of the line to be the effective modulus if the *R*^2^ of the linear regression was greater than 0.998. If not, a line was iteratively fit to a slightly smaller section of the curve, and the *R*^2^ requirement relaxed, until if the *R*^2^ value of the linear fit up to a strain of 0.005 was not greater than 0.945, the structure was assigned a modulus of 0. This is because, upon visual inspection, nearly all of the structures to which this applied lacked some internal connectivity, thus invalidating the results of the simulation. The modulus of 0 was intended to penalize these structures and indicate to the CNN surrogate model that they were undesirable.

### CNN and GA evaluation

CNNs are artificial neural networks commonly applied to classification or regression problems with image-based data^44,45^. The CNN was constructed using PyTorch, an open-source machine learning framework from Meta AI. It consisted of four convolutional layers, each followed by a ReLU (rectified linear unit) activation function and MaxPool (maximum pooling) layer. The outputs from the last MaxPool layer were flattened and followed by two fully connected layers, each similarly passed through ReLU activation functions, which finally output a single predicted value for each input image. This architecture was selected based on performance after exploring variations of such parameters as layer number, activation functions, and learning rate.

GAs perform global stochastic optimization by utilizing natural selection-based functions such as genetic crossover, mutation, and survival of the fittest over multiple generations of “evolution”^46^. The GA was developed based on the algorithm proposed via pseudo-code by refs. 47 and 48, especially for the random-weighted genetic algorithm (RWGA). In each generation of the GA, 2 “children” structures were generated with linear interpolation between “parent” structures, one “child” was generated with gene crossover, two more “children” were generated with point mutations of an existing “child” or “parent”, and two “pure” individuals were introduced. Then, three individuals were randomly selected for attrition, and the four least fit the remaining individuals were removed. The genetic algorithm was designed to emphasize exploration, however, our investigation of GA parameters was not exhaustive. Further variations on the GA may have enhanced structure optimization or latent space exploration. For further details regarding the CNN or GA, please see the code in the supplementary materials.

### Trabecular bone dataset

The trabecular bone images comprised SR-microCT scans of fresh-frozen bovine tibial condyle were taken at the Diamond-Manchester Imaging Branchline I13-2 at Diamond Light Source (UK) under proposal MG22575, see references for detail^49,50^. Images used to train StyleGAN were randomly selected from two-dimensional slices of five unique samples of trabecular bone.

### Supplementary Information

Supplementary Information

Description of Additional Supplementary Files

Supplementary Movie 1

Supplementary Movie 2

## Data Availability

The data that support the findings of this study, including training data, results, and source data for graphs, are available at https://github.com/lamm-mit/LeafGAN. Please consult the corresponding author with further questions or requests.

## Code availability

All open-source libraries used for image processing, neural network construction, coarse-grained simulations, etc., are available online.

OpenCV: https://opencv.org/

NumPY: https://numpy.org/

Trimesh: https://trimsh.org/

StyleGAN Encoder: https://github.com/pbaylies/stylegan-encoder

PyTorch: https://pytorch.org/

LAMMPS: https://www.lammps.org/

Custom code that supports the findings of this study, including StyleGAN training details, image processing code, simulation details, and CNN and GA code are available at https://github.com/lamm-mit/LeafGAN.
