# Supplementary material for: Nature-inspired architected materials using unsupervised deep learning
Source: Commun Eng. 2022 Nov 25;1:37. doi: 10.1038/s44172-022-00037-0 (PMC10955928; doi:10.1038/s44172-022-00037-0)
Supplement: Supplementary file 1 — Supplementary Information [file 44172_2022_37_MOESM1_ESM.pdf]

Supplementary Information

Manuscript: Nature-inspired architected materials using unsupervised deep learning

Authors: Sabrina Chin-yun Shen[1,2], Markus J. Buehler[1,3*]

Affiliations:
[1] Laboratory for Atomistic and Molecular Mechanics (LAMM), Massachusetts Institute of Technology, 77 Massachusetts Ave., Cambridge, MA 02139, USA

[2] Department of Materials Science and Engineering, Massachusetts Institute of Technology, 77 Massachusetts Ave., Cambridge, MA 02139, USA

[3] Center for Computational Science and Engineering, Schwarzman College of Computing, Massachusetts Institute of Technology, 77 Massachusetts Ave., Cambridge, MA 02139, USA

*mbuehler@mit.edu

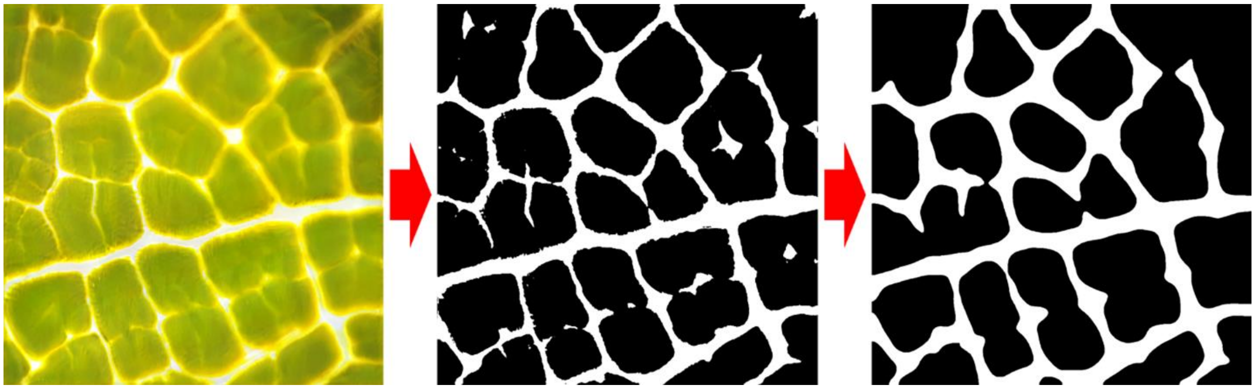

Supplementary Figure 1: Example of image processing from a leaf micrograph (left) to a smoothed image (center), and with islands removed (right)

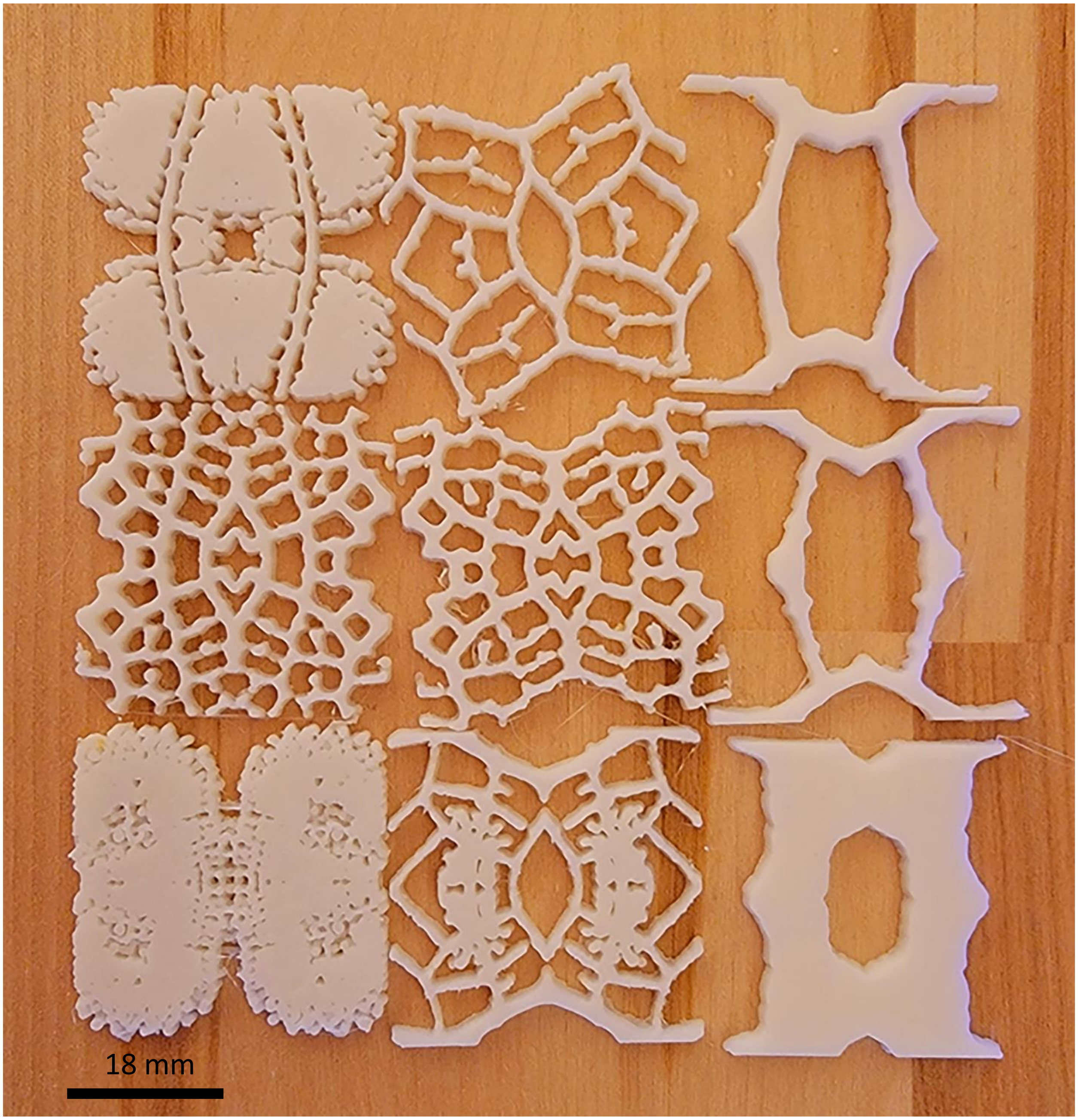

Supplementary Figure 2: Additional sample microstructures, implemented here as quasi-2D materials, generated from the model.

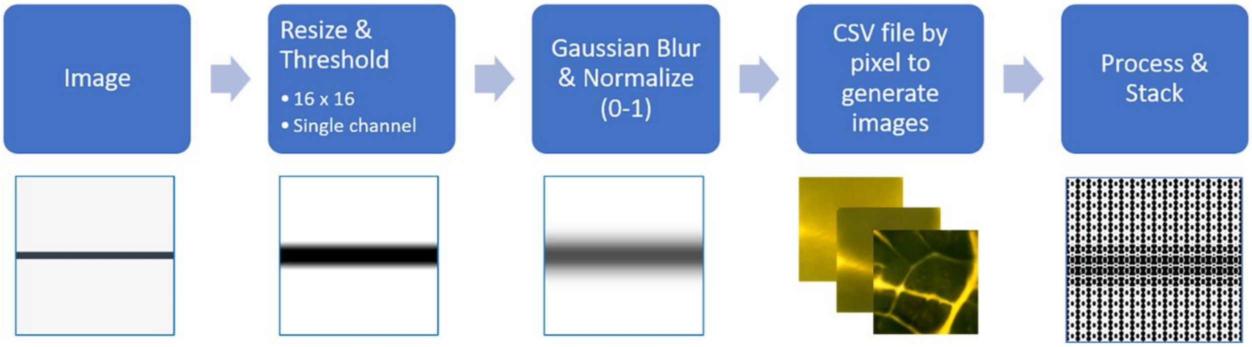

Supplementary Figure 3: Img2Architecture process used in this paper.
