# Supplementary material for: Nature-inspired architected materials using unsupervised deep learning
Source: Commun Eng. 2022 Nov 25;1:37. doi: 10.1038/s44172-022-00037-0 (PMC10955928; doi:10.1038/s44172-022-00037-0)
Supplement: Supplementary file 2 — Description of Additional Supplementary Files [file 44172_2022_37_MOESM2_ESM.pdf]

Description of Additional Supplementary Files

File name: Supplementary Movie 1

Description: Movement through latent space.

File name: Supplementary Movie 2

Description: Slicing animation, showing the internal structures of the architected material designed in Figure 6.
